# Viability of SARS‐CoV‐2 in faecal bio‐aerosols

**DOI:** 10.1111/codi.15181

**Published:** 2020-06-20

**Authors:** Jay Patel

**Affiliations:** ^1^ University of Leeds Leeds UK

*Dear Editor,*

I read with interest the rapid review by Gupta *et al*. [1] concerning the incidence and timing of faecal samples positive for severe acute respiratory syndrome coronavirus 2 (SARS‐CoV‐2) in patients with coronavirus disease (COVID‐19). The authors acknowledge insufficient evidence to support transmission via a faeco‐oral route and identify the need for further research regarding the viability of SARS‐CoV‐2 in the context of human faeces. However, it is equally pertinent to consider the viability of the virus in faecal bio‐aerosols generated by toilet plumes [2]. During the SARS outbreak, it was suggested that a major contributor to the rapid transmission of the initial 187 cases in Hong Kong was propagation by an aerosolization effect of SARS‐CoV‐1 in faeces with notably high viral load [3]. There is considerable variation in the stability of the novel coronavirus according to surface type, hence the need to replicate studies in the context of human faeces. Feasibly, fomites in the immediate environment of toilets may also accommodate viable SARS‐CoV‐2 from virus‐laden aerosols generated by toilet flushing. [4] If modelled in accordance with the inverse‐square law, the extent of viral dispersion from toilet plumes may be relatively far from the source. Data for the stability of SARS‐CoV‐2 on hospital lavatory fomites may warrant supplementary measures to mitigate the risk of potential fomite‐mediated nosocomial infection[5].

This consideration extends to the handling of faecal wastes discharged from hospitals and others sources. Sewage discharged from two hospitals in Beijing during the SARS outbreak was identified as positive for SARS‐CoV‐1 [6]. Using quantitative real‐time reverse transcription PCR methods, Wang and colleagues confirmed the presence of SARS‐CoV‐2 RNA in hospital inlets of a preprocessing disinfection sewage pool [7]. Although this review is currently dismissive of faeco‐oral transmission based on the available evidence, data in this area are rapidly evolving. Given the far‐reaching consequences that this would pose for the environment and risk of nosocomial infection, research into the viability of SARS‐CoV‐2 in faecal bio‐aerosols in addition to human faeces should be considered an urgent priority. Heightened care in the management of faecal wastes is an imperative intervention until these questions are answered.

## Funding

None.

## Conflicts of interest

I declare no competing interests.
